# Robustness Evaluation of Physical versus Virtual Bolus for Breast VMAT Using RegGAN‐Based Synthetic CT

**DOI:** 10.1002/pro6.70083

**Published:** 2026-08-12

**Authors:** Yong Sang, Lingke Kong, Jianan Wu, Junqin Lei, Enzhuo Quan, Qichao Zhou

**Affiliations:** ^1^ Department of Radiation Oncology National Cancer Center/National Clinical Research Center for Cancer/Cancer Hospital & Shenzhen Hospital Chinese Academy of Medical Sciences and Peking Union Medical College Shenzhen China; ^2^ Manteia Technologies Co., Ltd, 3001–3005 Xiamen City Fujian P. R. China

**Keywords:** cone beam CT, RegGAN, Robustness, Skin Involvement Breast Cancer, synthetic CT, Virtual Bolus, Volumetric modulated arc therapy

## Abstract

**Background:**

Volumetric modulated arc therapy (VMAT) for breast cancer with skin involvement requires a bolus to ensure adequate surface dose. Although both physical and virtual bolus methods are clinically established, their dosimetric robustness against interfraction anatomical variations remains underexplored. This study utilized deep learning–based synthetic CT (sCT) to evaluate the robustness of these two strategies.

**Methods:**

Ten patients with skin‐involved breast cancer treated with VMAT were retrospectively analyzed. Two plans were generated for each patient: plan_pb_ (physical bolus during simulation) and plan_vb_ (virtual bolus optimization). To evaluate robustness, pretreatment cone beam CT images from fractions 1, 6, and 11 were converted to sCT using a registration‐based generative adversarial network (RegGAN). Dose distributions were recalculated on these sCT to quantify deviations in the conformity index (CI), homogeneity index (HI), and volume percentage of the prescribed dose (*V*pd).

**Results:**

Initial planning showed no statistically significant differences between the two methods in target coverage or organs at risk sparing. In the robustness analysis performed on sCT, the virtual bolus method (plan_vb_) demonstrated statistically superior robustness for Vpd (*P* < 0.05), although the absolute magnitude of improvement was small (mean Vpd deviation: –0.8% vs –1.5%). The physical bolus method (plan_pb_) showed better robustness for CI.

**Conclusions:**

Using a RegGAN‐based evaluation framework, this study demonstrates that although both bolus strategies are dosimetrically comparable, the virtual bolus method provides marginally improved robustness in target dose coverage against daily anatomical variations.

## Graphical abstract

Utilizing RegGAN‐generated synthetic CTs, this study evaluates the dosimetric robustness of physical versus virtual bolus strategies in breast VMAT. Results indicate that the virtual bolus method provides comparable plan quality with superior robustness for target coverage homogeneity against daily anatomical variations.

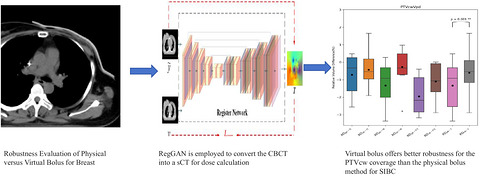

## 1 INTRODUCTION

Breast cancer is the most common malignancy, accounting for 23.8% of cancers in women, according to the latest statistics from the International Agency for Research on Cancer, with approximately 2.3 million new cases reported in 2022.^1^ Radiotherapy is a primary therapeutic modality for breast cancer. Postmastectomy radiotherapy (PMRT) has been shown to improve local control and overall survival, particularly in patients with node‐positive disease or locally advanced features.^2^, ^3^ For patients with skin‐involved breast cancer (SIBC), delivering an adequate dose to the skin surface often necessitates the use of a tissue‐equivalent bolus during treatment.^4^, ^5^

Although custom 3D‐printed boluses provide superior skin conformity and reproducibility,^6^, ^7^, ^8^ their widespread adoption remains limited because of logistical and workflow barriers, including extended fabrication times, the need for dedicated staffing for quality assurance, scalability challenges, and regulatory hurdles. Consequently, clinical practice predominantly involves placing a physical bolus on the patient's chest wall during CT simulation and reproducing this placement for each treatment fraction (Method 1). Although established, this workflow presents several challenges. Bolus application during simulation is time‐consuming. More importantly, it introduces a potential systematic error: air gaps or folds present during the single simulation scan are incorporated into the dose calculation; however, these imperfections may not be reproduced—or may differ—during daily treatment.

To address these limitations, a “virtual bolus” workflow has been adopted by some institutions (Method 2). In this approach, the patient undergoes simulation without a bolus. A “virtual” bolus structure with uniform thickness and density is then digitally generated on the body surface within the treatment planning system (TPS) for dose optimization. During treatment delivery, a physical bolus is placed on the patient to replicate the planned virtual structure. This method streamlines the simulation workflow and enables planning on an idealized surface free of random air gaps. Although this technique was originally developed for pseudo‐skin flash (i.e., treatment without a physical bolus),^9^, ^10^ its optimization principles also offer significant advantages for physical bolus planning. Optimization studies have suggested that defining a virtual bolus with specific parameters—commonly recommended as a 10–15 mm expansion and Hounsfield units (HU) of –400 to –600—can significantly enhance plan robustness against respiratory motion and anatomical deformation compared with physical bolus strategies.

However, a critical validation gap remains in this hybrid workflow. Although the virtual bolus plan assumes perfect contact, actual treatment involves a physical bolus with inevitable daily variations and air gaps. It remains unclear whether a plan optimized on a perfect virtual geometry is sufficiently robust when delivered to a real patient with an imperfect physical bolus, compared with the standard method of planning directly on the physical bolus. Most prior studies have relied on static phantom measurements or simulated rigid shifts, which fail to capture the complex, nonrigid anatomical changes (e.g., swelling and seroma reduction) that occur over the course of treatment.^11^ Assessing this robustness is technically challenging because the image quality of daily cone beam CT (CBCT) is typically insufficient for accurate dose calculation owing to scatter and shading artifacts.

To address this limitation, our study introduces a novel retrospective dose accumulation framework. We retrospectively analyzed patients with SIBC undergoing PMRT, comparing the physical and virtual bolus methods. Robustness was assessed by converting pretreatment CBCT images into synthetic CT (sCT) using a registration‐based generative adversarial network (RegGAN) framework^12^ and recalculating the dose distributions. This study enables a comprehensive, fraction‐by‐fraction evaluation of dosimetric robustness under simulated treatment conditions, thereby providing clinically relevant insights for decision‐making.

## 2 MATERIALS AND METHODS

### 2.1 Patients

**FIGURE 1:**
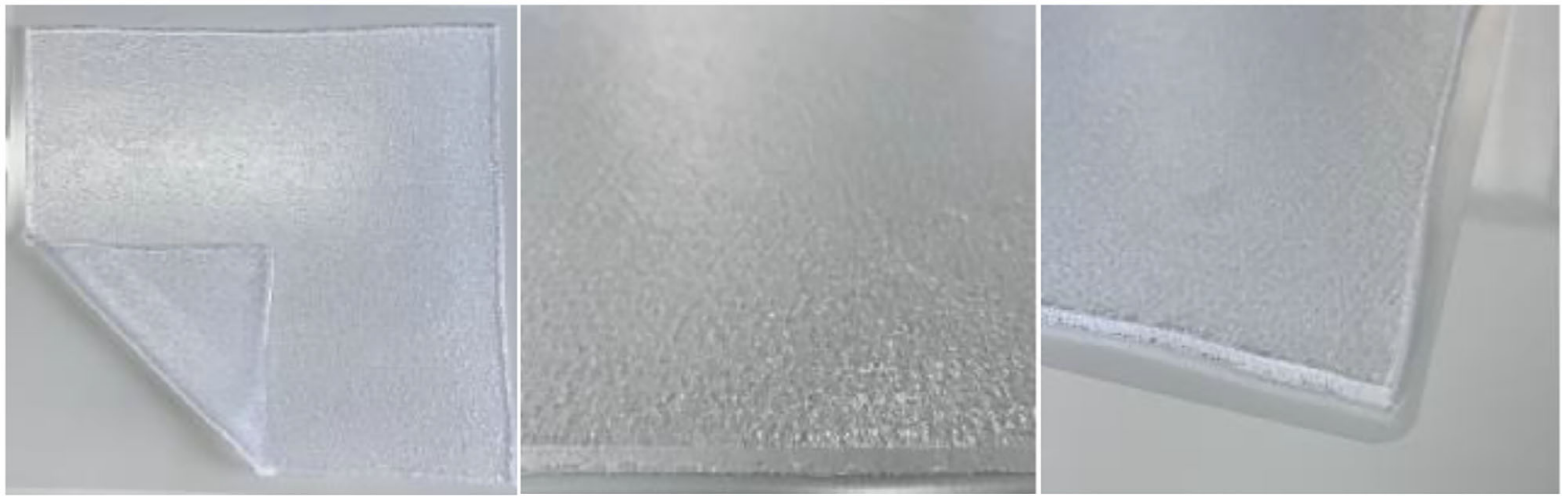
Physical bolus used in our department.

**FIGURE 2:**
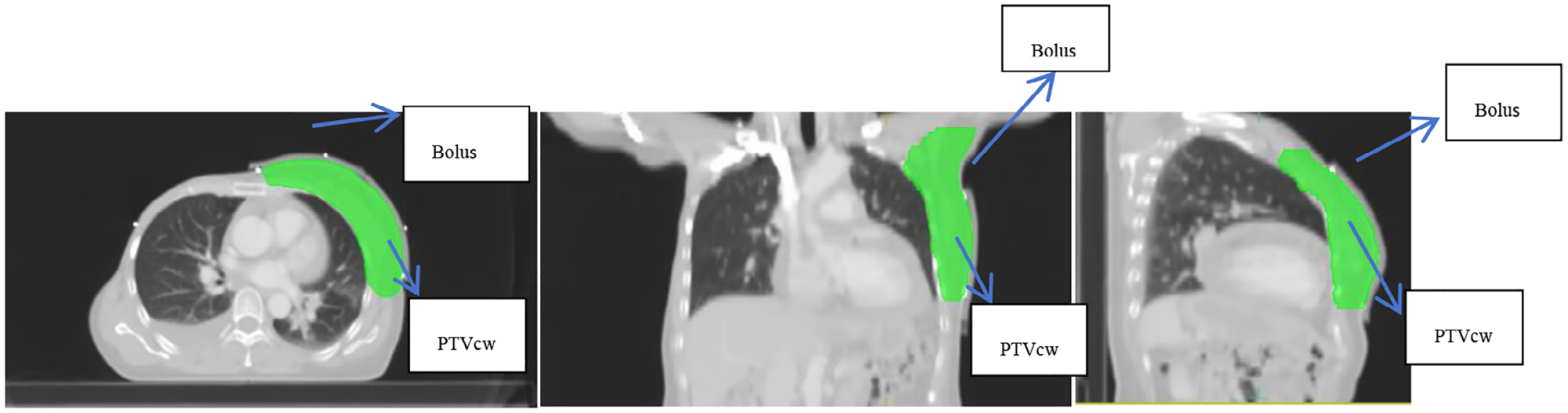
CT simulation image of a patient with an applied bolus. PTVcw refers to the chest wall target volume. “Bolus” indicates the physical bolus applied during CT simulation.

This study was approved by the Institutional Ethics Committee (Approval No. JS2024‐30‐1). We retrospectively included 10 patients with SIBC who completed treatment between May 2024 and May 2025. Among the 10 patients, 4 were classified as T3 and 6 as T4. Regarding nodal status, 3 patients were N0, 5 were N1, and 2 were N2; all patients were M0. One patient underwent CT simulation and treatment using the deep inspiration breath‐hold (DIBH) technique, whereas the remaining nine patients underwent both CT simulation and treatment under free‐breathing conditions because of poor reproducibility during DIBH training. During CT simulation, a conventional bolus (Gray Medical, Guangdong, China) was placed over the skin‐involved area. The bolus was composed of thermoplastic elastomer (TPE), with a physical density of 0.8 g/cc, a relative electron density of 0.8 compared with water, and a thickness of 5 mm (Figure 1). Our institution selected this TPE‐based bolus material because it conforms more effectively to skin contours than silicone materials with a density of 1 g/cc. Table 1 presents the baseline clinical characteristics of the included patients with SIBC. Among the 10 patients, 2 had chest wall PTV (PTVcw) as the target volume, whereas the remaining 8 had both PTVcw and supraclavicular PTV (PTVsc) as target volumes. The axilla and internal mammary nodes were not irradiated in any patient. Figure 2 shows a CT simulation image of a patient with the applied bolus.

**TABLE 1:** Baseline clinical characteristics of patients with SIBC (n = 10; all female).

	Age (years)	Volume of PTVcw (cc)	Volume of PTVsc (cc)
Mean	54.6	875	204
Median	53	815	212
Range	45–76	384–1442	140–251

### 2.2 Treatment planning

To ensure consistency in plan design, all selected patients with SIBC had their treatment plans redesigned by the same medical physicist. All plans were generated using the Monaco TPS (version 6.0; Elekta, Sweden). All cases included PTVcw and, where applicable, PTVsc, with a prescribed dose of 43.5 Gy delivered in 15 fractions.^13^ Treatment plans were optimized using volumetric modulated arc therapy (VMAT) with 6 MV flattening filter‐free beams. For left‐sided breast cancer, gantry rotation ranged from 300° to 180° and back, whereas for right‐sided cases, it ranged from 180° to 60° and back. Dose calculation was performed using the X‐ray voxel‐based Monte Carlo (XVMC) algorithm,^14^ with a calculation grid size of 0.3 cm and a statistical uncertainty of 1% per calculation.

Generation of plan_pb_: The plan_pb_ (physical bolus plan) was created by defining the external contour (External_1_) to include both the patient's body and the bolus as acquired during CT simulation. Respiratory motion was managed using a 2 cm auto‐flash margin in the TPS, which is standard practice at our institution for free‐breathing breast VMAT plans.^15^, ^16^, ^17^ This approach was selected for its practicality and seamless integration into our clinical workflow. The bolus was explicitly incorporated into the dose calculation, ensuring that the planned dose distribution accounted for its physical presence during treatment.

Generation of plan_vb_: The plan_vb_ (virtual bolus plan) was generated by mathematically deriving the external contour (External_2_) through Boolean subtraction of the bolus structure from External_1_, ensuring that the patient's skin geometry remained identical between the two planning scenarios. A 5 mm virtual bolus was then introduced to conform to the patient's skin surface, replicating the dimensions of the physical bolus in the lateral and craniocaudal directions. Critically, this method differs from standard skin flash techniques, which extend fluence into open air. In this approach, the virtual structure serves as an idealized optimization model, whereas a corresponding physical bolus is explicitly applied to the patient during treatment delivery to achieve the planned buildup. The Monaco XVMC algorithm calculates dose based on the relative electron density of materials compared with water; thus, both human tissues and bolus materials are represented by their respective relative electron density values in the dose calculation. This is achieved either through the Hounsfield unit (HU)–to–relative electron density conversion curve or by direct manual assignment of relative electron density values. Notably, directly assigned relative electron density values take precedence over those derived from the HU–to–electron density conversion curve. Therefore, the virtual bolus was assigned a relative electron density of 0.8 compared with water, identical to that of the physical bolus. A 2 cm auto‐flash margin was also applied to maintain consistency in respiratory motion management.

To ensure clinical validity, all plans were reviewed by experienced radiation oncologists. This approach ensured consistency in plan design and enabled a direct and meaningful comparison between the physical and virtual bolus plans while preserving clinical relevance.

### 2.3 Dosimetric evaluations

To evaluate the dosimetric differences between the generated plans, the following metrics were analyzed:

1. Conformity index (CI): This metric measures how well the prescribed dose conforms to the target volume. The formula is CI = TV_PV_ × TV_PV_ / (V_PTV_ × V_TV_), where V_TV_ is the volume covered by the prescribed isodose line, V_PTV_ is the volume of the PTV, and TV_PV_ is the target volume receiving the prescribed dose_._ A CI value closer to 1 indicates better conformity.^18^, ^19^ Because CI values calculated using this formula are less than 1, a higher value indicates better conformity.
2. Homogeneity index (HI): This metric evaluates dose uniformity within the target volume. The formula is HI = *D*_5%_ / *D*_95%_. A lower HI value indicates better homogeneity.^20^, ^21^
3. Organs at risk (OAR) metrics: These included the mean dose (*D*_m_) to the heart, ipsilateral lung, and contralateral breast; the volume percentage (*V*_5Gy_) of the ipsilateral and contralateral lungs; and the volume percentage (*V*_20Gy_) of the ipsilateral lung.

### 2.4 Application of planvb to physical bolus CT

To evaluate the dosimetric differences when applying plan_vb_ (virtual bolus plan) to CT images acquired with a physical bolus, the dose distribution was recalculated using the XVMC algorithm in Monaco, resulting in plan_vb‐pb_ (virtual bolus plan recalculated on physical bolus CT images). All dosimetric metrics outlined in Section 2.3 were analyzed for both plan_vb_ and plan_vb‐pb_. In addition, the volume percentage of the prescribed dose (*V*pd) within PTVcw, denoted as Vpd, was calculated. The results for each evaluation metric were reported for the 10 patients as median (min, max).

### 2.5 Conversion of CBCT to sCT

**FIGURE 3:**
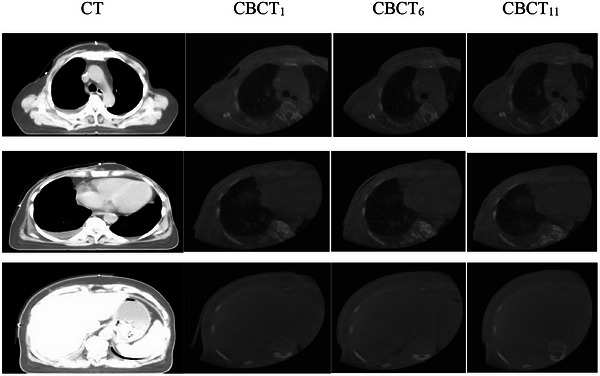
Comparison of the same axial plane among CT, CBCT_1_, CBCT_6_, and CBCT_11_ images from the same patient. CT denotes the CT simulation image. CBCT_1_ denotes the CBCT image acquired and registered before the first treatment fraction. CBCT_6_ denotes the CBCT image acquired and registered before the sixth treatment fraction. CBCT_11_ denotes the CBCT image acquired and registered before the eleventh treatment fraction.

**FIGURE 4:**
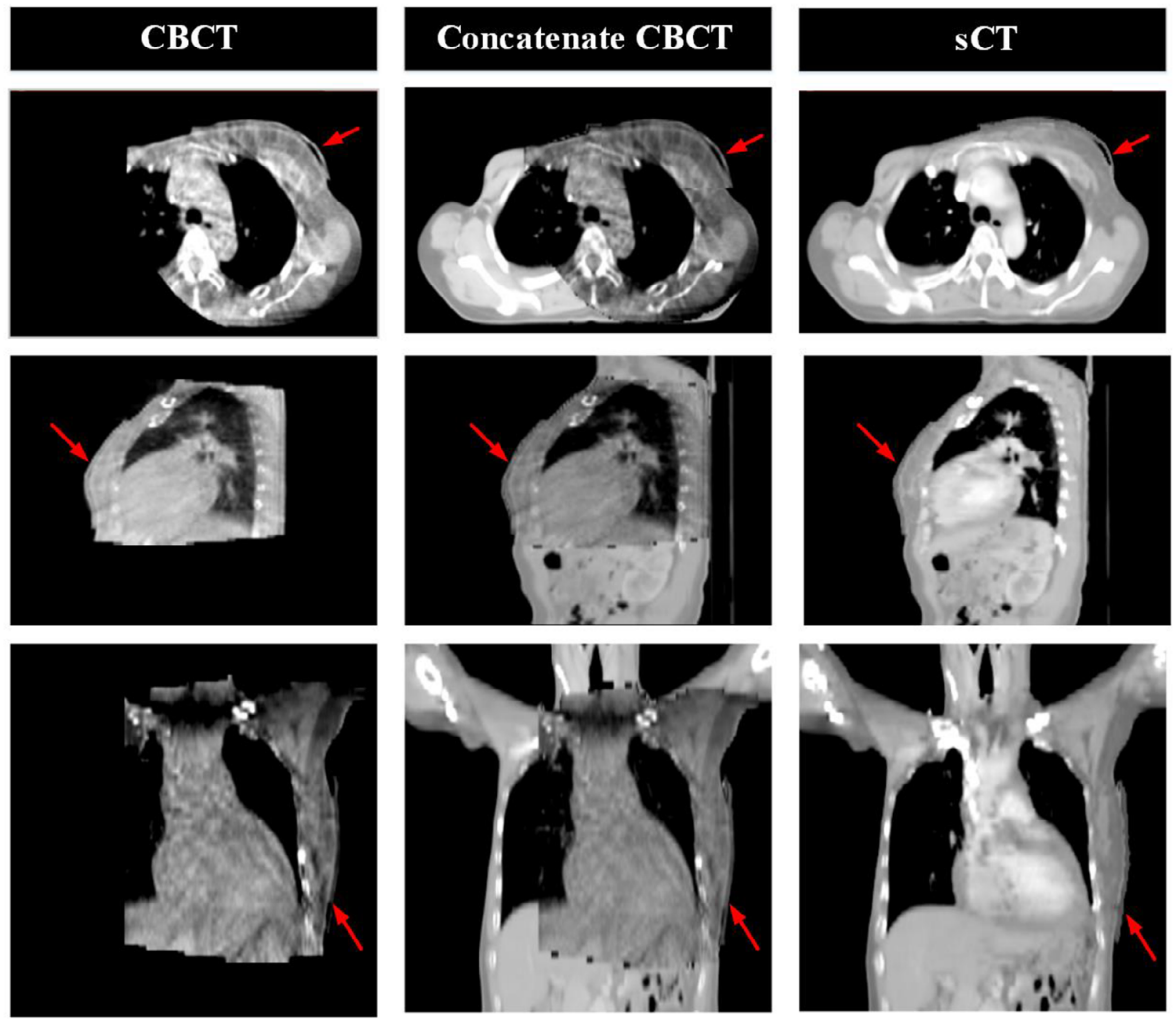
Effectiveness of converting CBCT to sCT. CBCT denotes the CBCT_1_ image acquired and registered before the first treatment fraction of patient 2 (Figure 7). Concatenated CBCT denotes the fused image of CBCT and CT images. sCT denotes the synthetic CT used for dose calculation.

To evaluate robustness, pretreatment cone beam computed tomography CBCT images acquired at the first, sixth, and eleventh fractions (CBCT_1_, CBCT_6_, and CBCT_11_) for each patient (total n = 30) were converted to sCT using a RegGAN‐based framework.^12^, ^22^, ^23^ Detailed descriptions of the RegGAN architecture, training procedure, loss functions, and validation metrics—mean absolute error (MAE), normalized MAE, peak signal‐to‐noise ratio (PSNR), and structural similarity index measure—are provided in Supplementary Materials S1 and S2. Briefly, RegGAN integrates a U‐Net generator and a patch‐based discriminator with a registration component to improve anatomical alignment. Performance validation of this model demonstrated high quantitative fidelity, with a mean MAE of 39.9 ± 12.1 HU and a PSNR of 27.0 ± 2.2 dB. Contours from the planning CT were rigidly propagated to the sCT images for dose recalculation. Figure 3 presents representative axial images, and Figure 4 illustrates the conversion performance.

It is acknowledged that minor residual setup errors may persist after online CBCT guidance. In this study, all fractions adhered to the clinical tolerance of ≤ 1 mm shifts and ≤ 1° rotations. These residual errors were intentionally retained in the dose accumulation process to faithfully represent the actual geometric uncertainty of treatment delivery rather than an idealized setup.

### 2.6 Robustness evaluation in the treatment position

To evaluate the robustness of plan_pb_ and plan_vb_, the dose distributions during actual treatment were reproduced by recalculating the plans in the Monaco TPS using the XVMC algorithm on sCT_1_, sCT_6_, and sCT_11_ images converted from the pretreatment CBCT. The recalculated plans were designated as plan_pb‐t1_, plan_pb‐t6_, and plan_pb‐t11_ (physical bolus plans in the treatment position for the first, sixth, and eleventh fractions, respectively) and plan_vb‐t1_, plan_vb‐t6_, and plan_vb‐t11_ (virtual bolus plans in the treatment position for the first, sixth, and eleventh fractions, respectively). The dose metrics described in Section 2.3 were used to evaluate these plans. For a given dosimetric evaluation metric X, the relative deviation (RD) for planpb was defined as RD_pb_ = (Xplan_pb‐t_ − Xplan_pb_) / Xplan_pb_ × 100%, and the RD for planvb was defined as RD_vb_ = (Xplan_vb‐t_ − Xplan_vb_) / Xplan_vb_ × 100%.

### 2.7 Statistical analysis

For plan_pb_, plan_vb_, plan_vb‐pb_, RD_vb‐pb_, plan_pb‐t1_, plan_pb‐t6_, plan_pb‐t11_, plan_vb‐t1_, plan_vb‐t6_, plan_vb‐t11_, RD_pb‐t1_, RD_pb‐t6_, RD_pb‐t11_, RD_vb‐t1_, RD_vb‐t6_, and RD_vb‐t11_, the results are presented as median (min, max) (n = 10). For RD_pb‐t_ (denoting all relative deviations of the physical bolus, including RD_pb‐t1_, RD_pb‐t6_, and RD_pb‐t11_) and RD_vb‐t_ (denoting all relative deviations of the virtual bolus, including RD_vb‐t1_, RD_vb‐t6_, and RD_vb‐t11_), the results are presented as mean ± standard deviation (n = 30). To investigate differences between plan_pb_ and plan_vb_, a paired, two‐tailed, nonparametric Wilcoxon signed‐rank test was used to compare paired data. This test was selected because of the small sample size and nonnormal data distribution. To evaluate differences between the recalculated plans on sCT (plan_pb‐t_ and plan_vb‐t_), reflecting the robustness of Method 1 and Method 2, a repeated‐measures analysis of variance (ANOVA) was performed to compare RDpb‐t and RDvb‐t. This approach accounts for clustering of 30 fraction‐level observations within 10 patients across three time points and properly addresses the correlation among repeated measurements. A *P* value < 0.05 was considered statistically significant. Statistical analyses were performed using SPSS version 19.0 (SPSS Inc., Chicago, IL, USA).

Given the limited patient cohort, effect sizes (partial η^2^) and 95% confidence intervals (CIs) for the mean differences were calculated for all endpoints within the repeated‐measures ANOVA framework. Furthermore, to distinguish statistical significance from clinical relevance, a minimal clinically important difference (MCID) was defined a priori. Although standard global dosimetric guidelines typically accept an overall treatment delivery uncertainty of 3–5%, this study specifically isolated a single planning variable (bolus placement strategy) while holding the dose calculation algorithm and delivery platform constant. To prevent an isolated planning variance from compounding with downstream delivery errors and thereby exceeding global safety limits, a deliberately strict and conservative MCID of 1% was adopted for target coverage $\left(V_{pd}\right)$ and the HI. Therefore, deviations of < 1% were considered clinically equivalent, whereas differences exceeding this threshold were classified as clinically meaningful.

## 3 RESULTS

### 3.1 Dosimetric evaluation of plan_pb_ and plan_vb_

**FIGURE 5:**
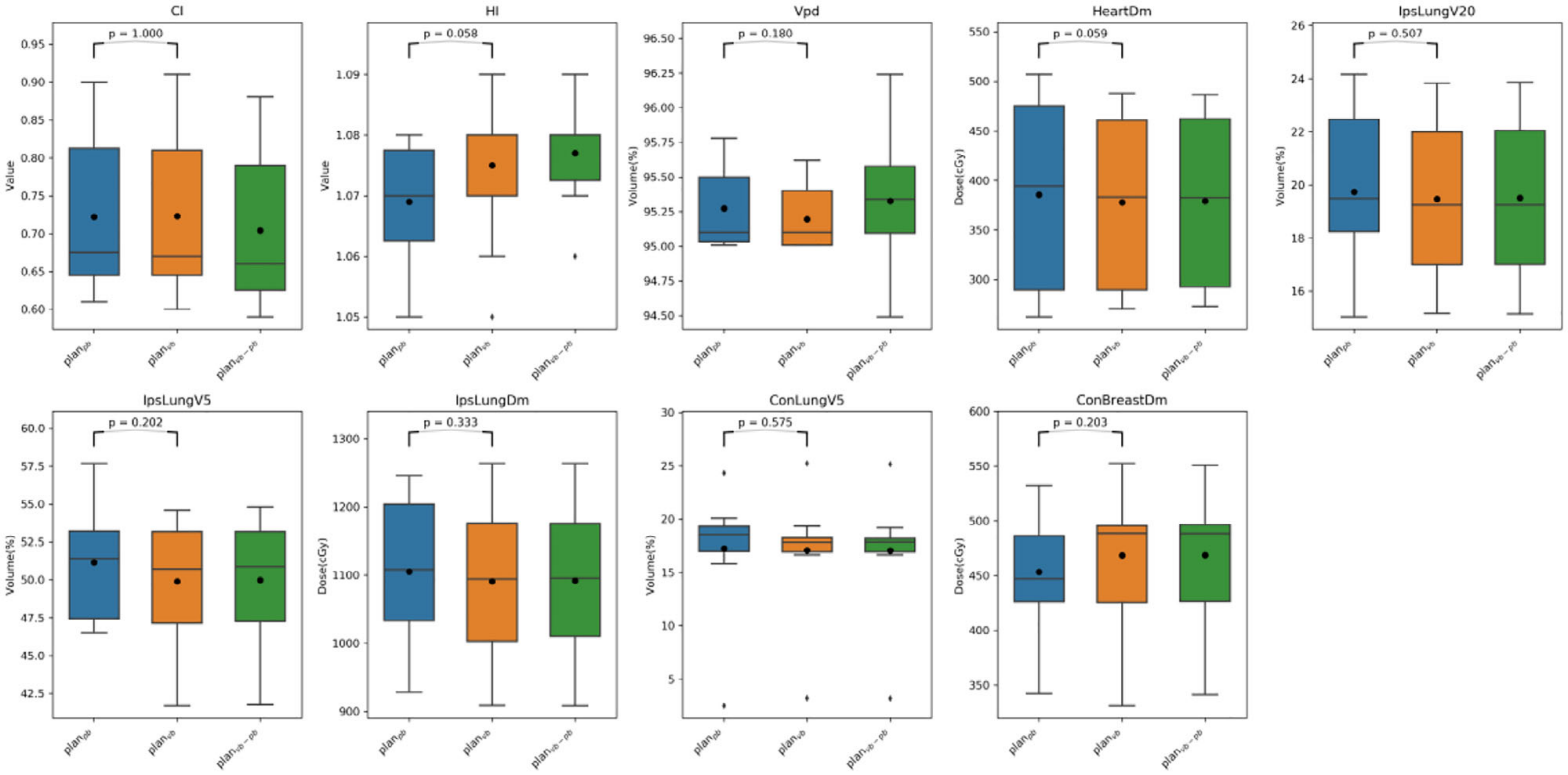
Comparison of plan_pb_, plan_vb_, and PTV_vb‐pb_ for PTVcw and OARs. CI: conformity index; HI: homogeneity index; *V*pd: volume percentage of the prescribed dose; *D*_m_: mean dose; Ips: ipsilateral; Con: contralateral; RD: relative difference; RD_vb‐pb_ = (X_planvb‐pb_ – X_plan_vb_) / X_plan_vb_ × 100%, where X represents the value of a specific patient evaluation metric; RDM_vb‐pb_: mean value of RD_vb‐pb_ for the 10 patients.

Figure 5 and Table S3 (in the Supplementary Materials section) present a comparison between plan_pb_ and plan_vb_ using the paired, two‐tailed, nonparametric Wilcoxon signed‐rank test. The results indicate no statistically significant differences in CI, HI, or *V*pd for PTVcw. Similarly, no statistically significant differences were observed across all OAR metrics.

### 3.2 Dosimetric evaluation of plan_vb_ and plan_vb‐pb_

The dosimetric comparison between plan_vb_ and plan_vb‐pb_ (virtual bolus plan recalculated on the physical bolus CT) is also presented in Figure 5 and Table S3. The mean relative difference (RDM) for most evaluation metrics was less than 1%, except for the CI of PTVcw. The *V*pd of PTVcw, a critical parameter for target coverage, exhibited an RDM of only 0.1% across the 10 patients. In the worst case among the 10 patients, the *V*pd changed minimally from 95.01% to 94.49%, indicating a negligible dosimetric impact when applying a virtual bolus plan to a physical bolus CT.

### 3.3 Evaluation of plan_pb_ and plan_pb‐t_, and plan_vb_ and plan_vb‐t_

**FIGURE 6:**
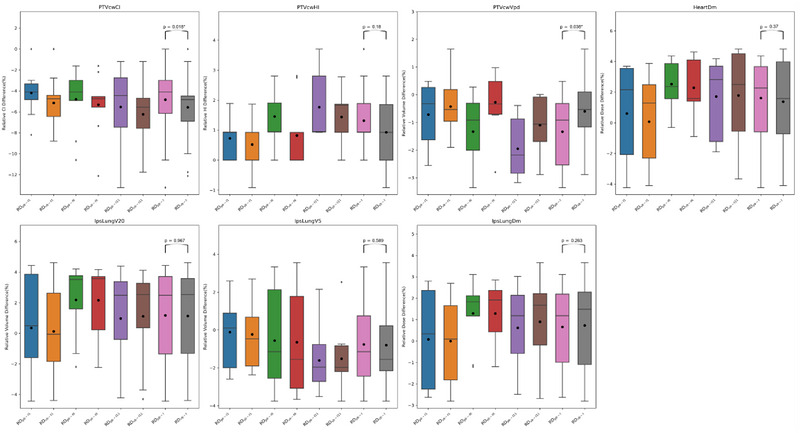
Robustness comparison between plan_pb‐t_ and plan_vb‐t_ for the 10 patients. CI: conformity index; HI: homogeneity index; *V*pd: volume percentage of the prescribed dose; *D*_m_: mean dose; Ips: ipsilateral; Con: contralateral; RD: relative difference; RD_pb‐t_ = (X_planpb‐t_ − X_plan_pb_) / X_plan_pb_ × 100%; RD_vb‐t_ = (X_planvb‐t_ − X_planvb_) / X_planvb_ × 100%, where X represents the value of a specific patient evaluation metric. plan_pb‐t1_, plan_pb‐t6_, and plan_pb‐t11_ denote the physical bolus plans in the treatment position for the first, sixth, and eleventh fractions, respectively, plan_vb‐t1_, plan_vb‐t6_, and plan_vb‐t11_ denote the virtual bolus plans in the treatment position for the first, sixth, and eleventh fractions, respectively. RD_pb‐t_ denotes all relative deviations of the physical bolus, including RD_pb‐t1_, RD_pb‐t6_, and RD_pb‐t11_; RD_vb‐t_ denotes all relative deviations of the virtual bolus, including RD_vb‐t1_, RD_vb‐t6_, and RD_vb‐t11_.

**FIGURE 7:**
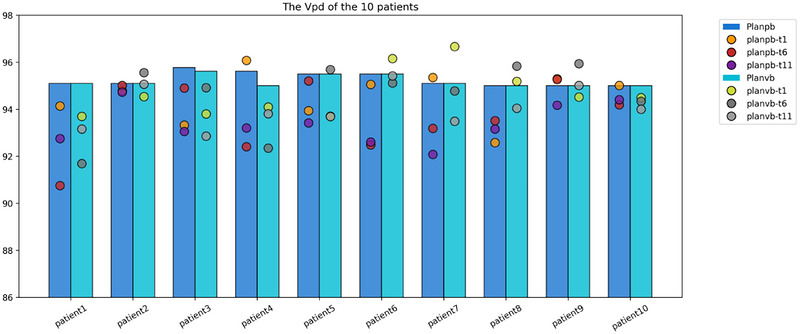
Vpd values for plan_pb_, plan_pb‐t1_, plan_pb‐t6_, plan_pb‐t11_, plan_vb_, plan_vb‐t1_, plan_vb‐t6_, and plan_vb‐t11_ across the 10 patients. *V*pd: volume percentage of the prescribed dose for PTVcw; plan_pb_: physical bolus plan; plan_vb_: virtual bolus plan, plan_pb‐t1_, plan_pb‐t6_, and plan_pb‐t11_ denote the physical bolus plans in the treatment position for the first, sixth, and eleventh fractions, respectively, plan_vb‐t1_, plan_vb‐t6_, and plan_vb‐t11_ denote the virtual bolus plans in the treatment position for the first, sixth, and eleventh fractions, respectively.

Figure 6 and Table S4 (in the Supplementary Materials section) summarize the dosimetric differences between plan_pb_ and plan_pb‐t_, as well as between plan_vb_ and plan_vb‐t_ (plans recalculated on sCT). The largest RD was observed in the CI of PTVcw, with a maximum of −10.2% for plan_pb‐t_ and −10.1% for plan_vb‐t_, and mean values of −4.7% and −5.6%, respectively. For the most clinically relevant parameter, *V*pd of PTVcw, the maximum relative deviations were −4.6% for plan_pb‐t_ and −3.6% for plan_vb‐t_, and the mean relative deviations were −1.5% for plan_pb‐t_ and −0.8% for plan_vb‐t_. Figure 7 presents a bar chart comparing *V*pd across all 10 plans.

Figure 6 and Table S4 present the robustness comparison between plan_pb‐t_ and plan_vb‐t_ based on 30 fraction‐level observations (10 patients × 3 time points). A repeated‐measures ANOVA was performed to account for clustering of observations within patients. A significant main effect of method was observed for CI (*F* = 8.230, *P* = 0.018) and Vpd (*F* = 5.928, *P* = 0.038), indicating a statistically significant difference in robustness between the two methods for these metrics. Plan_pb‐t_ (Method 1) demonstrated better robustness for CI, whereas plan_vb‐t_ (Method 2) demonstrated better robustness for *V*pd. No significant main effect of method was observed for HI (*F* = 2.109, *P* = 0.180), suggesting comparable robustness between the two methods for target homogeneity. For all OAR metrics, no significant main effects of method were observed (all *P* > 0.05), indicating that both methods provide similar robustness for OARs. Table 2 summarizes the effect sizes and 95% CIs for all endpoints. Despite the large effect sizes observed for CI (partial *η* ^2^ = 0.478) and *V*pd (partial *η* ^2^ = 0.397), the absolute mean differences were small (0.9% and 0.7%, respectively) and fell below the predefined clinical equivalence threshold of 1%. The Bonferroni‐corrected 95% CI for Vpd included zero, indicating that the statistical evidence for a difference was not conclusive after correction for multiple comparisons.

**TABLE 2:** Comparison of dosimetric robustness between physical and virtual bolus methods.

Metric	Preferred method	Mean difference (VB − PB)	95% CI for difference	*P* value	Partial *η* ^2^
CI	PB	−0.9%	[−1.7%, −0.2%]	0.018	0.478
HI	NSD	−0.3%	[−0.7%, 0.2%]	0.180	0.190
*V*pd	VB	0.7%	[0.0%, 1.3%]	0.038	0.397
Heart *D*_m_	NSD	−0.2%	[−0.6%, 0.3%]	0.370	0.090
Ips Lung *V*_20_	NSD	−0.007%	[−0.4%, 0.3%]	0.967	0.000
Ips Lung *V*_5_	NSD	−0.1%	[−0.3%, 0.2%]	0.589	0.034
Ips Lung *D*_m_	NSD	0.1%	[−0.1%, 0.2%]	0.263	0.137

Abbreviations: CI: conformity index; HI: homogeneity index; *V*pd: volume percentage of the prescribed dose; *D*_m_: mean dose; Ips: ipsilateral; PB: physical bolus; NSD: no significant difference; VB: virtual bolus.

## 4 DISCUSSION

Unlike previous studies that focused solely on static planning comparisons, our use of RegGAN‐generated sCT enabled a fraction‐by‐fraction evaluation of robustness. Using this CBCT‐to‐sCT conversion method, the robustness comparison between plan_pb_ and plan_vb_ (resulting in plan_pb‐t_ and plan_vb‐t_, respectively) demonstrated that the CI of PTVcw calculated on sCT images acquired during treatment exhibited a smaller reduction for the physical bolus plan than for the virtual bolus plan. This finding indicates that the physical bolus plan provides greater robustness in target dose conformity. For the Vpd of PTVcw calculated on sCT, the virtual bolus plan exhibited a smaller reduction compared with the physical bolus plan, indicating that the virtual bolus plan provides greater robustness in target coverage despite variations in bolus placement and the presence of air gaps.

The comparable robustness between the two plans can be partially attributed to the imperfect conformity of the conventional bolus to the skin surface in patients with breast cancer. During CT simulation, the bolus is placed over the skin‐involved area; however, the air gap between the bolus and the skin may not remain consistent between simulation and actual treatment. Even when CBCT is performed before treatment, this inconsistency is difficult to eliminate. Moreover, patients with irregular or uneven skin surfaces may experience greater variability in bolus adherence, further affecting reproducibility.

These observations are consistent with previous research findings. Using head phantoms, Kong et al.^24^ demonstrated that 3D‐printed customized boluses achieved smaller air gaps and superior dosimetric parameters compared with commercial boluses^25^, highlighting the critical role of bolus–skin conformity in treatment accuracy. In clinical practice, air gaps between commercial boluses and irregular skin surfaces can exceed 1 cm, making it difficult to replicate the same conditions from CT simulation to treatment delivery. In Figure 3 of this study, a noticeable difference in the air gaps between the bolus and the skin surface on the simulation CT and CBCT can be observed. Additionally, minor variations in bolus placement by radiation therapists between CT simulation and actual treatment may also contribute to this variability. Moreover, despite CBCT‐based registration, residual differences in patient positioning between CT simulation and treatment can further affect the results.

Another contributing factor is respiratory motion and the inherent softness of breast tissue, which may lead to changes in the target volume and surrounding anatomical structures between localization and treatment. These anatomical variations may further explain the comparable robustness observed in this study. The DIBH technique may help mitigate the impact of this factor. Because of these influences, even within the same patient, the RD_pb‐t_ of Vpd for PTVcw varied across treatment fractions, as did the RD_vb‐t_, as illustrated in Figure 7.

In addition, the XVMC algorithm in Monaco has been shown to achieve acceptable dosimetric agreement in heterogeneous phantoms and to model tissue–air interface effects more realistically than traditional collapsed‐cone convolution methods.^14^ This study also highlights a key practical advantage of Method 2. Whereas Method 1 requires placement of a physical bolus during simulation, which can be time‐consuming and workflow‐constraining, Method 2 allows addition of a virtual bolus during treatment planning, thereby offering greater flexibility and ease of implementation. Therefore, we recommend the virtual bolus method (Method 2) for patients with SIBC receiving VMAT with a conventional bolus, as it effectively meets target dose requirements while providing superior robustness in target coverage and improving clinical workflow efficiency.

The TPE bolus material used in this study (relative electron density 0.8; physical density ≈ 0.83 g/cc) was selected primarily for its superior pliability and conformity to irregular chest wall geometry compared with rigid standard boluses. Although water‐equivalent materials (density ≈ 1.0 g/cc) remain the clinical standard, recent literature underscores that air gaps—which are more prevalent with rigid or thick bolus sheets—are a more critical source of surface dose reduction than minor variations in material density.^6^, ^8^ Although the absolute dosimetric values in this study reflect the 0.8 g/cc material, the relative robustness advantage of the virtual strategy—namely, elimination of air gaps—remains applicable regardless of the specific bolus density used.

It is important to note that although some studies recommend lower densities (e.g.,−400to−600HU) for “pseudo‐skin flash” techniques in which no physical bolus is applied, our workflow involves application of a physical TPE bolus during treatment. Therefore, the virtual bolus density was matched to that of the physical bolus (0.8 g/cc) to ensure accurate dose calculation, rather than using the lower‐density air‐mixture values typically applied in flash optimization.

To evaluate the 30 paired datasets (3 fractions × 10 patients), we calculated effect sizes (partial *η* ^2^) and 95% CIs. However, it is crucial to interpret these statistically significant findings within a clinical context. Although the initial repeated‐measures ANOVA suggested potential differences in target coverage ($V_{pd}\;\mathrm{partial}\;\;\eta^{2}=0.397$), subsequent Bonferroni correction indicated that these differences were no longer statistically significant (95% CI included zero). Furthermore, the absolute magnitude of the mean difference (0.7%) fell below the predefined 1% MCID threshold. These findings confirm that the two methods are dosimetrically equivalent. Although the virtual bolus workflow offers a measurable improvement in consistency, its primary clinical advantage lies in operational efficiency—namely, eliminating the need for physical bolus placement during simulation and reducing patient discomfort—rather than in a decisive difference in dosimetric outcomes.

Regarding the applicability of these findings to other fractionation schedules and motion management techniques, it is important to note that the hypofractionated regimen (43.5 Gy in 15 fractions) used in this study represents a conservative scenario for robustness evaluation. Conventional fractionation (e.g., 50 Gy in 25 fractions) involves a greater number of treatment fractions, which may further average out random daily setup errors. Consequently, the robustness observed in this study is likely transferable—if not superior—to longer treatment courses. Similarly, although this study primarily evaluated free‐breathing delivery, the virtual bolus method is expected to remain robust during DIBH treatments. Because DIBH effectively minimizes respiratory motion and chest wall deformation compared with free breathing, the geometric stability of the virtual bolus would theoretically improve, provided that intrafraction breath‐hold reproducibility is strictly monitored.

This study has several limitations. First, because of the limited scan range of CBCT, missing regions were supplemented by merging them with simulation CT images, which may have introduced uncertainties in dose calculation, particularly for beam angles passing through these regions. Although only a small subset of arc angles passed through the stitched regions (primarily in the posterior thorax), the potential dosimetric impact was mitigated by the relative anatomical stability of the posterior chest wall and the robustness of the registration algorithm in these low‐gradient regions. Consequently, any residual uncertainties in the patched anatomy were considered negligible compared with the primary setup errors affecting the target volume. Second, even after CBCT‐based positional correction, residual setup errors may still have resulted in suboptimal image registration, thereby compromising conversion accuracy. Third, contours were propagated from the planning CT using rigid registration. Although this approach reflects the standard non‐adaptive image‐guided radiotherapy workflow, it does not account for soft‐tissue deformation (e.g., swelling), which highlights the potential benefit of adaptive replanning. Fourth, variations in bolus placement techniques and manual handling by different radiation therapists may have influenced the study results. However, rather than representing a confounding bias, these variations reflect the inherent clinical reality of physical bolus application. Our results demonstrate that the physical bolus method is intrinsically sensitive to such interoperator variability (e.g., air gap formation), whereas the virtual bolus workflow effectively eliminates this operator‐dependent variable, thereby providing a more consistent and reproducible dosimetric outcome. Finally, although post hoc statistical analysis confirmed adequate power for the evaluated metrics, this study was limited to a cohort of ten patients and a sampling frequency of three fractions (1, 6, and 11) per treatment course. Although this sampling strategy captures the general temporal trend of anatomical changes, it may miss random daily variations (e.g., peak seroma swelling) occurring between sampled time points. Future studies using automated batch‐processing pipelines should include larger, multi‐institutional cohorts and full‐course daily dosimetric accumulation to capture the complete spectrum of interfraction variability.

## 5 CONCLUSION

By leveraging RegGAN‐based sCT for accurate dose accumulation, this study provides a novel evaluation of bolus strategy robustness. We found that the virtual bolus method offers comparable robustness in target coverage compared with physical bolus placement, likely because of the elimination of variable air gaps inherent in physical bolus application. However, given that the absolute dosimetric advantage is marginal and falls below the predefined clinically meaningful threshold, the primary clinical benefit of the virtual bolus method lies in its operational efficiency and improved patient comfort. Therefore, the virtual bolus method represents a safe and operationally efficient alternative for VMAT in the treatment of SIBC.

## AUTHOR CONTRIBUTIONS

YS designed the study, collected the data, and drafted the manuscript. JW was responsible for data collection. KL was responsible for converting CBCT to sCT. JL was responsible for verification of CBCT–CT registration. EQ and QZ designed the study and revised the manuscript. All authors read and approved the final manuscript.

## FUNDING

This work was partly supported by the Shenzhen Science and Technology Program (JCYJ20240813151606009) and the Sanming Project of Medicine in Shenzhen (No. SZSM202211030).

## CONFLICT OF INTEREST STATEMENT

The authors declare that they have no competing interests.

## ETHICS STATEMENT

Not applicable.

## CONSENT FOR PUBLICATION

Not applicable.

## Supporting information



Supporting Information

## Data Availability

All data generated or analyzed during this study are included in this published article.
