# Supplementary material for: Robustness Evaluation of Physical versus Virtual Bolus for Breast VMAT Using RegGAN‐Based Synthetic CT
Source: Precis Radiat Oncol. 2026 Aug 12;10(3):296–306. doi: 10.1002/pro6.70083 (PMC13463262; doi:10.1002/pro6.70083)
Supplement: Supplementary file 1 [file PRO6-10-296-s001.docx]

**Supplementary materials**

**S1 Training Details**

All experiments were conducted on 64-bit Ubuntu with 96 GB RAM and an Nvidia Titan RTX (24 GB) using a PyTorch environment. Images were normalized to [-1, 1]. Training used the Adam optimizer (learning rate 1e-4, β₁=0.5, β₂=0.999), batch size 1, and weight decay 1e-4 for 200 epochs. Loss-function weights are summarized in Table S1.

Table S1. Weights for different loss functions

| Loss | L_adv_ | L_corr_ | L_smooth_ |
| --- | --- | --- | --- |
| Weight | 1 | 20 | 10 |

**S2 Evaluation of image conversion results**

We evaluated model performance using MAE, NMAE, PSNR, and SSIM (Table S2). MAE measures overall HU deviation; NMAE normalizes the HU range; PSNR reflects sensitivity to large errors; and SSIM assesses structural preservation. Together, these metrics provide a comprehensive evaluation.

Table S2. Evaluation of image conversion results

|  | MAE | NMAE | PSNR | SSIM |
| --- | --- | --- | --- | --- |
| mean±std | 39.935±12.063 | 0.013±0.004 | 26.987±2.186 | 0.863±0.035 |

Figure S1 illustrates model performance by comparing CBCT, CT, sCT, and their error map. The error map shows CT–sCT differences, with red indicating larger discrepancies and blue indicating smaller ones, providing a visual assessment of sCT accuracy relative to CT.

Figure S1. Comparison images of CBCT, CT, sCT, and error maps for different patients

Table S3. Dosimetric comparison of plan_pb_ and plan_vb_ (median(min,max))

|  | PTVcw CI | PTVcw HI | PTVcw Vpd(%) |
| --- | --- | --- | --- |
| plan_pb_ | 0.675  (0.610,0.900) | 1.07  (1.05,1.08) | 95.1  (95.01,95.78) |
| plan_vb_ | 0.670  (0.600,0.910) | 1.08  (1.05,1.09) | 95.1  (95.01,95.62) |
| Z | 0.000 | -1.897 | -1.342 |
| p | 1.000 | 0.058 | 0.180 |

No statistically significant differences (p > 0.05) were observed for Heart Dmean, Ipsilateral Lung V5/V20/Dmean, Contralateral Lung V5, or Contralateral Breast Dmean. CI: conformity index, HI: homogeneity index, Vpd: volume percentage of the prescribed dose.

Table S4. The robustness comparison between plan_pb-t_ and plan_vb-t_. Data presented as Mean **±** SD relative deviation (RD) from the initial plan across all evaluated fractions.

|  | PTVcw  CI | PTVcw  HI | PTVcw  Vpd(%) | Heart  D_m_(Gy) | Ips Lung  V_20_(%) | Ips Lung  V_5_(%) | Ips Lung  D_m_(Gy) |
| --- | --- | --- | --- | --- | --- | --- | --- |
| **RD_pb-t_** | **-4.7%±3.0%** | **1.3%±0.9%** | **-1.5%±1.3%** | **1.6%±2.4%** | **1.2%±3.0%** | **-0.8%±2.1%** | **0.6%±1.9%** |
| **RD_vb-t_** | **-5.6%±2.8%** | **1.0%±1.0%** | **-0.8%±1.2%** | **1.5%±2.8%** | **1.2%±3.0%** | **-0.8%±2.0%** | **0.7%±2.0%** |

Abbreviations: CI: conformity index; HI: homogeneity index; Vpd: volume percentage of prescribed dose; Dm: mean dose. Note: RD calculated as (Plan_treatment_ - Plan_initial_) / Plan_initial_ × 100%.
